# Interactions between endocarditis-derived *Streptococcus gallolyticus *subsp. *gallolyticus *isolates and human endothelial cells

**DOI:** 10.1186/1471-2180-10-78

**Published:** 2010-03-16

**Authors:** Tanja Vollmer, Dennis Hinse, Knut Kleesiek, Jens Dreier

**Affiliations:** 1 Institut für Laboratoriums- und Transfusionsmedizin, Herz- und Diabeteszentrum Nordrhein-Westfalen, Bad Oeynhausen, Germany

## Abstract

**Background:**

*Streptococcus gallolyticus *subsp. *gallolyticus *is an important causative agent of infective endocarditis (IE) but the knowledge on virulence factors is limited and the pathogenesis of the infection is poorly understood. In the present study, we established an experimental *in vitro *IE cell culture model using EA.hy926 and HUVEC cells to investigate the adhesion and invasion characteristics of 23 *Streptococcus gallolyticus *subsp. *gallolyticus *strains from different origins (human IE-derived isolates, other human clinical isolates, animal isolates). Adhesion to eight components of the extracellular matrix (ECM) and the ability to form biofilms *in vitro *was examined in order to reveal features of *S. gallolyticus *subsp. *gallolyticus *endothelial infection. In addition, the strains were analyzed for the presence of the three virulence factors *gtf*, *pilB*, and *fimB *by PCR.

**Results:**

The adherence to and invasion characteristics of the examined *S. gallolyticus *subsp. *gallolyticus *strains to the endothelial cell line EA.hy926 differ significantly among themselves. In contrast, the usage of three different *in vitro *models (EA.hy926 cells, primary endothelial cells (HUVECs), mechanical stretched cells) revealed no differences regarding the adherence to and invasion characteristics of different strains. Adherence to the ECM proteins collagen I, II and IV revealed the highest values, followed by fibrinogen, tenascin and laminin. Moreover, a strong correlation was observed in binding to these proteins by the analyzed strains. All strains show the capability to adhere to polystyrole surfaces and form biofilms. We further confirmed the presence of the genes of two known virulence factors (*fimB*: all strains, *gtf*: 19 of 23 strains) and demonstrated the presence of the gene of one new putative virulence factor (*pilB*: 9 of 23 strains) by PCR.

**Conclusion:**

Our study provides the first description of *S. gallolyticus *subsp. *gallolyticus *adhesion and invasion of human endothelial cells, revealing important initial information of strain variability, behaviour and characteristics of this as yet barely analyzed pathogen.

## Background

Viridans streptococci are the most important pathogens responsible for native valve infective endocarditis (IE) in non-drug-addicted patients [1]. However, *Streptococcus gallolyticus *subsp. *gallolyticus*, formerly referred to as *Streptococcus bovis *biotype I, a member of group D streptococci, was estimated to be the causative agent in 24% of streptococcal endocarditis [2]. *S. gallolyticus *subsp. *gallolyticus *belongs to the *S. bovis*-complex including different species frequently isolated from humans and animals (*S. bovis*, *S. gallolyticus*, *S. infantarius, S. equinus*, *S. alactolyticus*). The taxonomic classification of this group of streptococci was often revised. However, at the beginning of this decade, Schlegel *et al. *proposed the reclassification of *S. bovis *biotype I as *S. gallolyticus *subsp. *gallolyticus *based on genetic, physiologic and phylogenetic perceptions [3], which was recently confirmed in a large comprehensive study [4]. Therefore, the highest priority regarding the clinical significance must refer to *S. gallolyticus *subsp. *gallolyticus *instead of *S. bovis*. Particularly in Southern Europe, the proportion of endocarditis caused by group D streptococci increased over the recent years [5,6]. Hoen *et al. *documented that 58% (France), 9.4% (other European countries) and 16.7% (USA) of streptococcal IE cases were caused by *S. bovis *[6]. *S. gallolyticus *subsp. *gallolyticus *is a normal inhabitant of the human gastrointestinal tract and numerous reports, referring to *S. bovis*, demonstrated an association between IE and gastrointestinal neoplasia, which were in most cases colonic adenoma or carcinoma [7-9] as well as liver disease [10,11]. Either the underlying colonic disease or an altered hepatic function may promote the bacterial translocation during the initial phase of infection [10].

Pathogenesis and several virulence factors have been examined for viridans streptococci, yet the knowledge of similar mechanisms for *S. gallolyticus *subsp. *gallolyticus *is limited. Studies examined the adhesion of animal isolates from pigeons to immobilized matrix proteins [12], and characterized virulence-associated surface proteins [13-15]. Recently, Sillanpää *et al. *observed a difference in adherence to distinct host extracellular matrix (ECM) proteins of endocarditis-derived *S. gallolyticus *subsp. *gallolyticus *isolates [2]. Until now, analogue mechanisms of human isolates regarding the adhesion to or invasion of endothelial cells, as well as defined virulence genes are unknown. Viridans streptococci have been shown to adhere to human endothelial cells *in vitro *[16,17] and numerous host cell factors and bacterial components have been identified as possible virulence factor candidates in other streptococci [18]. For example, a group of streptococcal genes encoding several adhesins (*fimA*, *fimB*, *ssaB*, *scaA*, *psaA*) play important roles in the development of IE [19-21]. It has also been shown that *pilB *contributes to adherence to endothelial cells in groupB streptococci and over-expression leads to increased virulence in rats [22,23].

Glycosyltransferases (*gtf*), which are responsible for the synthesis of glucans, are known to be major cell surface proteins involved in adherence of *Streptococcus gordonii *to human umbilical vein endothelial cells (HUVECs) *in vitro *[24]. Glycosyltransferases are further involved in the adhesion to human endothelial cells [24] and modulate cellular cytokine induction in IE [25,26]. Biofilm formation *in vitro *is also strongly influenced by the amount of Gtf produced by *S. mutans *[27,28]. The role of biofilm formation in IE remains open, with some studies reporting a lack of association [29,30] and other studies proposing a considerable importance [31]. Endothelial matrix proteins, such as collagen, fibronectin, vitronectin, fibrinogen and laminin, have also been shown to serve as receptors for bacterial surface proteins which promote bacterial adherence to host cells [32-37].

In the present study, an *in vitro *cell culture model was established to analyze the capability of human S. *gallolyticus *subsp. *gallolyticus *endocarditis isolates to adhere to and invade endothelial cells in comparison to non-endocarditis associated strains. Furthermore, adherence to immobilized ECM proteins and the ability to form biofilms *in vitro *was examined to determine a possible association with endocarditis virulence. Isolates were further analyzed for the presence of the virulence associated genes *fimB*, *gtf *and *pilB*.

## Methods

### Bacterial strains and growth conditions

Bacterial strains listed in Table 1 were obtained from the American Type Culture Collection (ATCC, LGC Promochem GmbH, Wesel, Germany), the Deutsche Sammlung von Mikroorganismen und Zellkulturen GmbH (DSMZ, Braunschweig, Germany) or were previously isolated from patients' specimens at our hospital. All clinical isolates used in this study were characterized in our microbiological laboratory by standard methods. In addition, molecular genetic strain identification was performed by sequencing analysis of the superoxide-dismutase gene (*sodA*) as described previously [38]. Bacteria were grown on tryptone soya (TS) or brain heart infusion (BHI) agar plates (Oxoid Ltd., Cambridge, UK) and isolated colonies were used as inocula in TS or BHI medium. Determination of the bacterial titer was performed by plating on TS agar. For simplification of readability, *S. gallolyticus *subsp. *gallolyticus *is consecutively abbreviated to *S. gallolyticus*.

**Table 1:** List of strains analyzed in this study with source, *sodA *GenBank accession numbers, distribution of virulence gene sequences detected by PCR and occurrence of intestinal abnormalities.

*S. gallolyticus *strain	Sod A GenBank acc. no.	Source	*gtf*^1^	*pilB*^2^	*fimB*^3^	*intestinal abnormalities*
DSM 16831	FJ617226	feces, koala	+	-	+	not applicable
DSM 13808	FJ617227	sapropel	+	+	+	not applicable
Isolate 12932	FJ042703	heart valve culture, IE patient	+	-	+	nondistinctive tumour of the mesenteric root
Isolate 00718	FJ617228	heart valve culture, IE patient	-	-	+	irregular-shaped mucosa erythema Bulbus duodeni without oedema
Isolate 10288	FJ151353	heart valve culture, IE patient	+	+	+	clinical data not available
Isolate 03080	FJ151354	heart valve culture, IE patient	+	+	+	engrained rectal carcinoma
Isolate 10672	FJ151355	heart valve culture, IE patient	+	+	+	clinical data not available
Isolate 06718	FJ151356	heart valve culture, IE patient	+	+	+	pea-sized colon polyp with mucosa breakdown
Isolate 07849	FJ151357	heart valve culture, IE patient	+	-	+	inconspicuous gastroscopy and colonoscopy
Isolate 21702	FJ151358	heart valve culture, IE patient	+	+	+	swelling of intestinal wall in terms of chronic colon ischemia
ATCC 49475	FJ617229	clinical isolate of unknown origin	+	-	+	not applicable
ATCC 49147	FJ617230	clinical isolate of unknown origin	+	-	+	not applicable
Isolate 134257	FJ151359	heart valve culture, IE patient	+	-	+	inconspicuous gastroscopy and colonoscopy
Isolate 05950	FJ151360	heart valve culture, IE patient	+	+	+	clinical data not available
Isolate 13366	FJ151361	heart valve culture, IE patient	+	+	+	inconspicuous gastroscopy and colonoscopy
Isolate K6236	FJ151362	blood culture, IE patient	+	-	+	clinical data not available
Isolate AC6860	FJ617231	human clinical isolate	+	+	+	clinical data not available
Isolate AC1016	FJ617232	human clinical isolate	+	-	+	clinical data not available
Isolate AC1135	FJ617233	human clinical isolate	-	-	+	clinical data not available
Isolate AC1181	FJ151364	human clinical isolate	-	-	+	clinical data not available
Isolate AC1242	FJ151365	human clinical isolate	-	-	+	clinical data not available
Isolate AC7070	FJ617234	human clinical isolate	+	-	+	clinical data not available
Isolate AC6872	FJ151367	human clinical isolate	+	-	+	clinical data not available

^1 ^gene coding for glycosyltransferase, ^2 ^gene coding for pilus-associated protein,

^3 ^gene coding for fibrinogen-binding protein

### Cell culture

EA.hy926 cells were derived from the ATCC (Manassas, USA) and cultured in Dulbecco's modified essential medium (DMEM, Gibco, Invitrogen, Karlsruhe, Germany) containing 1% L-glutamine (200 mM), supplemented with 10% fetal calve serum (Biowest, Nuaillé, France) and 1% antibiotic/antimycotic solution (100×, Biowest). Cells were grown at 5% CO_2 _and 37°C in a humidified atmosphere and were used before passage 10 for all experiments. Cryopreserved pooled HUVECs were purchased from Lonza (Basel, Switzerland) and grown routinely in EGM-2 medium (EGM-2 BulletKit, Lonza) at 5% CO_2 _and 37°C in a humified atmosphere. Cell culture dishes were previously coated with collagen I (Sigma, Steinheim, Germany). Cells were utilized before passage five for all experiments. EA.hy926 and HUVEC cells were detached using 10% trypsin-EDTA (Lonza). For the adherence and invasion assays, cells were transferred after trypsination to 6-well culture plates and cultivated to confluence. Before infection with bacteria, cells were washed three times with Dulbecco's phosphate buffered saline (DPBS, Gibco).

### Cell adherence and invasion assay

The assay was performed as described previously with some modifications [39,40]. Briefly, bacterial cultures were harvested at late-log phase, washed once with DPBS and diluted in fresh cell culture medium without antibiotics. To analyze dose-dependent effects on adhesion and invasion, two millilitres of bacterial suspensions with inocula between 10^2 ^and 10^7 ^CFU/mL were added to each well containing a monolayer of EA.hy926 or HUVEC cells, respectively. For the comparison of adhesion and invasion efficacy of different *S. gallolyticus *strains, cells were infected with 1 - 9 × 10^5 ^CFU/mL. The 6-well plates were incubated for 2 h at 37°C and 5% CO_2 _to facilitate cell infection. For the adherence assay, the cell monolayer was washed five times with DPBS, and endothelial cells were trypsinized and lysed with 1 mL 10% Trypsin-EDTA in distilled water. Trypsin-reaction was stopped with an equal volume of cell culture medium without antibiotics. 100 μL from each well were plated onto TS agar and incubated overnight at 37°C. For the invasion assay, the monolayer was washed three times with DPBS. Two millilitres of cell culture medium supplemented with 1% antibiotic/antimycotic solution and 100 μg/mL gentamicin (Gibco) were added to each well. The 6-well plates were incubated for another 2 h at 37°C and 5% CO_2 _to kill extracellular and surface-adherent bacteria. Afterwards, the monolayers were washed three times with DPBS and bacteria were quantified as described for the adherence assay. Assays were performed in duplicate and repeated twice. For comparative reasons, isolate 21702 was used as an internal assay control in every assay. Antibiotic efficacy of the invasion assay was tested for all strains with concentrations of 10^7 ^CFU/mL in pure cell culture medium, confirming that no viable bacteria were present after 2 h incubation (data not shown).

### Mechanical stretch

Cultures of EA.hy926 were subjected to cyclic tension using a FlexCell vacuum system (FlexCell, Dunn Laboratories, Hillsborough, USA). Cells were cultured on BioFlex culture plates (FlexCell) coated with collagen I in a humidified atmosphere with 5% CO_2 _at 37°C for 72 h. Afterwards cultures were stretched by 10% with a frequency of 1 Hz in a square wave pattern for another 24 h. EA.hy926 from the same preparation and cultured without mechanical stretch were used as controls. Stretched cells and controls were infected immediately after completion of mechanical stretch as described above.

### Biofilm assay

The biofilm assay used in this study was performed as described previously [30] with the following modifications: absorbance was measured using the GENios Plate Reader (Tecan Deutschland GmbH, Crailsheim, Germany) at 450 nm (total bacterial growth) and 550 nm (crystal violet (CV), biofilm formation). Each strain was assayed in quintuplicate.

### ECM assay

96 well microtiter plates were coated with 10 μg/mL fibrinogen (human plasma, Sigma). Microtiter plates precoated with collagen I, collagen II, collagen IV, fibronectin, laminin, tenascin and vitronectin were purchased from Chemicon (Millipore, Schwalbach, Germany). Wells coated with BSA were used as negative controls and values were subtracted. Late-log-phase cultures of bacteria were inoculated into 100 μL BHI medium (Oxoid) and incubated on pre-coated wells without agitation for 2 h at 37°C. Subsequently, wells were washed twice with DPBS and dried for 20 min at 60°C. In parallel, bacteria were plated onto BHI agar and incubated overnight at 37°C. Attached bacteria were stained with 100 μL of 0.4% CV at room temperature for 45 min. Wells were rinsed five times with PBS and air dried. CV was solubilized in 100 μL ethanol (99%), and the absorbance was measured at 550 nm. Each strain was assayed in quadruplicate for the different ECM proteins.

### PCR analysis

Bacteria were harvested from overnight grown cultures and bacterial DNA was extracted using the QIAamp DNA Blood Kit (protocol D, Qiagen, Hilden, Germany) according to the manufacturer's instructions. PCR primers were designed to amplify the known virulence factors of *S. gallolyticus fimB *and *gtf *and to amplify a homolog of the *pilB *gene identified in *S. suis *(Table 2). DNA amplification was carried out in 0.2 mL tubes containing 45 μL reaction mix and 5 μL DNA extract. The reaction mix consisted of 1× HotMaster *Taq *buffer including 2.5 mM MgCl_2_, 200 μM of each dNTP, 100 nM of each primer and 1.25 U of HotMaster *Taq *DNA polymerase (5 Prime, Inc., Gaithersburg, USA). The PCR conditions were as follows: initial denaturation at 94°C for 5 min, followed by 30 cycles of denaturation at 95°C for 30 s, PCR-product specific annealing temperature (Table 2) for 60 s and extension at 72°C for 60 s, followed by a final elongation for 10 min at 72°C. PCR products were sequenced for identification as described previously [41].

**Table 2:** Primer sequences and PCR conditions.

Primer	Oligonucleotide sequence (5'-3')	Nucleotide positions*	Annealing temperature	Amplicon length	Genbank accession no.
fimB-550F	GGTAAGTGATGGTATTGATGTC	550-571	45	347	AY321316
fimB-875R	GTGTTCCTTCTTCCTCAGTATT	875-896			
gtf-F	GGTGAGACTTGGGTTGATTC	2049-2068	54	496	AB292595
gtf-R	GCTCTGCTTGAACAACTGGA	2525-2544			
pilB-385F	AAGGGACGAGGGCTCTAC	120017-120034	58	339	CP000408
pilB-722R	ACCCAATTCCAACATACG	120373-120356			

*positions according to the respective Genbank accession no.

### Statistical analysis

Statistical analysis was performed using One-way-ANOVA, the Mann-Whitney-U-test and the student's t-test where appropriate. Multiple testing correction was performed using the Bonferroni method. Normality testing of all data sets for Gaussian distribution was performed using the Kolmogorov-Smirnov test. We used Spearman correlation coefficients to assess correlations between variables. P values < 0.01 were considered significant. All values are given as mean values (± SD). Statistical analysis was performed using GraphPad Prism 4.0 software (GraphPad Software, San Diego, CA, USA).

## Results

### Identification of virulence genes and occurrence of intestinal abnormalities

All strains analyzed in this study were identified as *S. gallolyticus *by sequencing analysis of the *sodA *gene (GenBank accession no. Table 1). Table 1 displays the distribution of the analyzed *S. gallolyticus *virulence genes *fimB*, *gtf *and *pilB *among 23 different strains. The known virulence gene *fimB *was detected in all analyzed strains, whereas four strains showed no positive PCR signal for *gtf*. The occurrence of a partial sequence homolog of the *pilB *gene, originally identified in *S. suis*, was proven in 9 strains of *S. gallolyticus *(GenBank accession no. for *S. gallolyticus *partial *pilB *sequence: FJ555059). Sequencing analysis confirmed the gene as *pilB *with a high similarity of 98% to *S. suis pilB*. PCR screening results were confirmed by Southern-Blot analysis with total genomic DNA and Digoxigenin-labeled PCR products (data not shown).

Analysis of the corresponding patient information of eight isolates revealed two patients exhibiting colonic malignancies, three patients with intestinal abnormalities and three patients without evidence of intestinal abnormalities (Table 1). For the other eleven human clinical isolates, patient data was not available because isolates were obtained from other institutes and repeatedly characterized in our microbiological laboratory.

### Adhesion to and invasion of EA.hy926 cells

**Figure 1:**
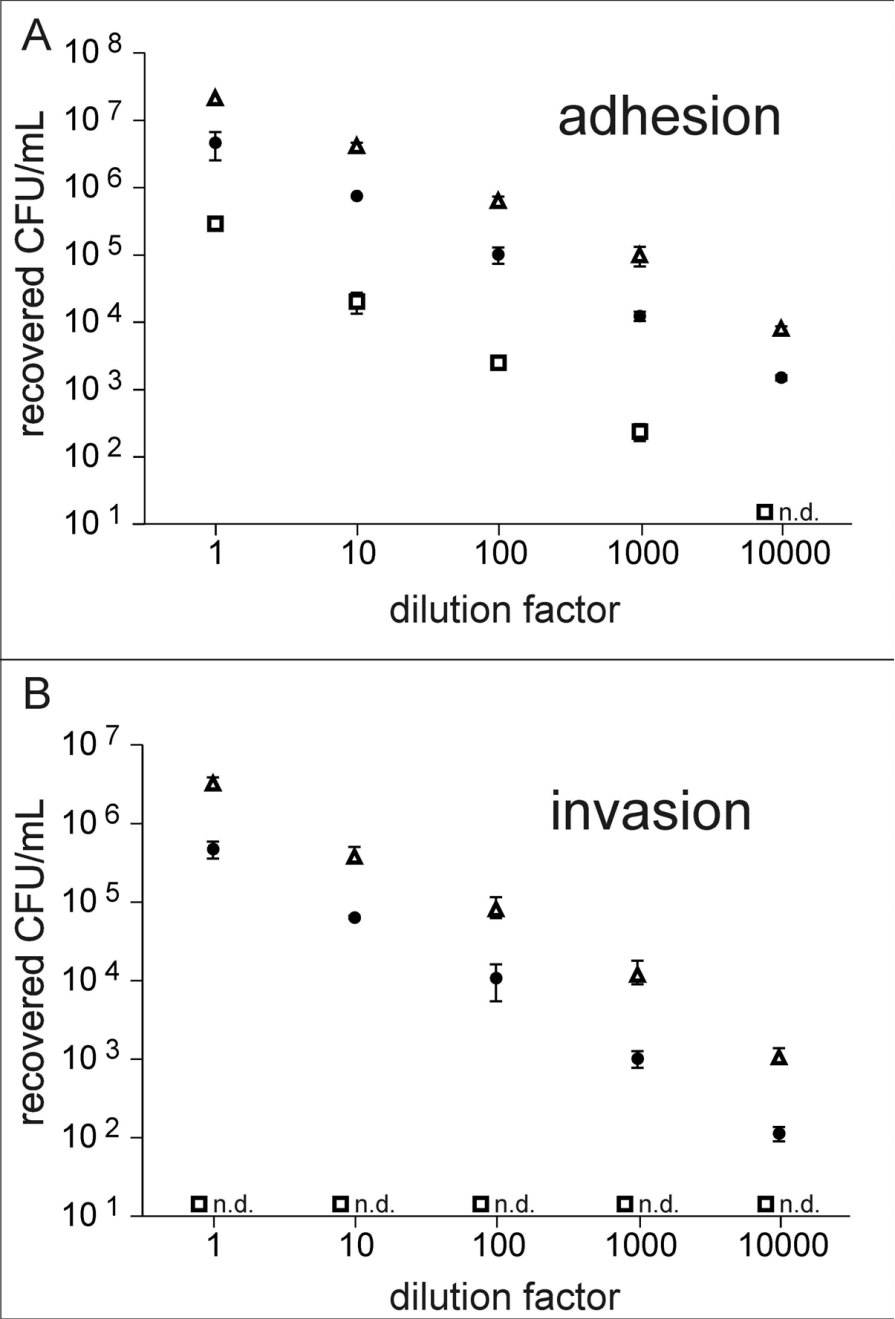
**Dose response analysis of *S. gallolyticus *adhesion to and invasion of EA.hy926 cells**. (A) Adhesion, (B) Invasion. Cells were incubated with decreasing concentrations of three different *S. gallolyticus *strains (white triangle: isolate 05950, black dot: isolate 21702, white square: DSM 16831), as described in Material and Methods. Error bars indicate standard deviations, n.d.: not detectable.

**Figure 2:**
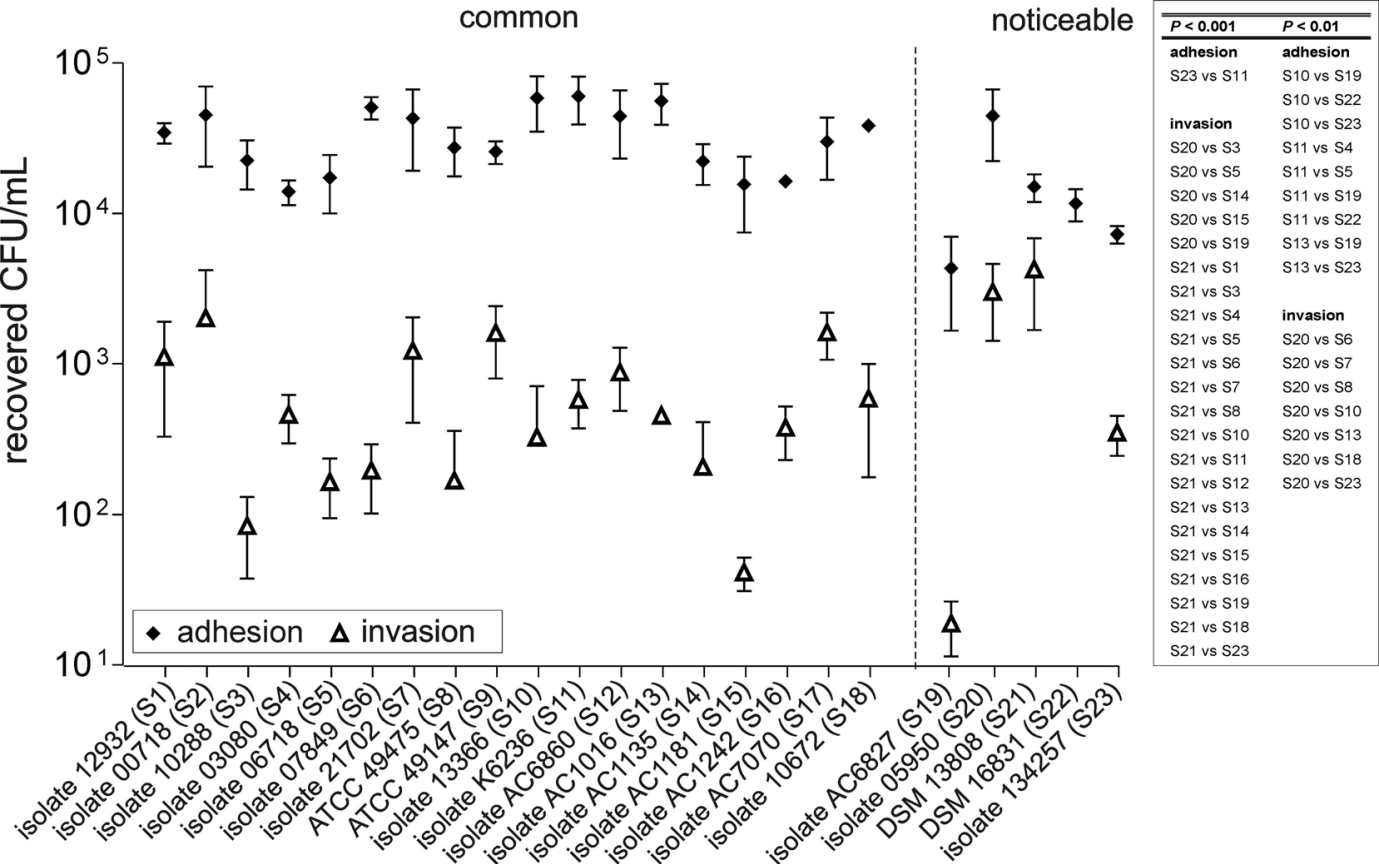
**Adhesion and invasion characteristics of different *S. gallolyticus *strains to EA.hy926 cells**. Displayed are the factorized adhesion to and invasion characteristics of 23 different *S. gallolyticus *strains (calculated to 1 × 10^5 ^CFU/mL) after 2 h infection of EA.hy926 cells. The *dashed vertical *line indicates the separation of "common" and "noticeable" relations between adhesion and invasion. Error bars indicate standard deviations. Results of statistical analysis of individual strains are arranged in tabular form.

All strains started to grow after 3 h of incubation in DMEM at 37°C and 5% CO_2 _at the earliest (data not shown). Therefore, incubation time for adhesion was determined to 2 h to avoid false-high titers as a result of bacterial growth kinetics. Three strains representing different adherence and invasion potentials, namely strain DSM 16831 (low adhesion, no invasion), isolate 21702 (intermediate adhesion and invasion) and isolate 05950 (high adhesion and invasion), were chosen to exemplify the dose-dependent effects on adhesion and invasion to EA.hy926 cells (Fig. 1). The proportion of adhesive and invasive bacteria did not increase using higher bacterial concentrations, with both, the adhesiveness and the invasiveness of the different bacteria showing a linear progress. Remarkably, strain DSM 16831 did not have the potential to invade cells, even when higher bacterial concentrations were used for infection. Subsequently, all *S. gallolyticus *strains were compared regarding their adhesion and invasion characteristics to EA.hy926 cells (Fig. 2). As a result of the observed linear progress and for strain comparability the initial inocula were calculated to 1 × 10^5 ^CFU/mL, and consequently adhesion and invasion values were factorized. Generally, all the *S. gallolyticus *strains analyzed were able to adhere to EA.hy926 endothelial cells (range 10^3^-10^4 ^recovered CFU/mL) and significant differences were observed among the investigated strains (repeated measures anova, *P *< 0.0001). Consideration of the individual strains revealed that isolates 13366, K6236 and AC1016 presented the most frequently significances (Fig. 2). With the exception of strain DSM 16831, which was excluded in further statistical analysis regarding invasion characteristics, all *S. gallolyticus *strains also had the capacity to invade EA.hy926 cells (range 10^1 ^- 10^3 ^recovered CFU/mL) with significant differences (repeated measures anova, *P *< 0.0001). A closer look on variation between individual strains disclosed, that the potential of invasion of the two strains DSM 13808 and isolate 05950 demonstrated numerous significances overall (DSM 13808: 17 strains, *P *< 0.001; isolate 05950: five strains, *P *< 0.001 and seven strains *P *< 0.01, Fig. 2). Correlation analysis of adherence and invasion showed a strong correlation for all strains (Spearman rank correlation coefficient r = 0.673, *P *= 0.0003). A considerable difference in the adherence or invasion potential of endocarditis isolates and reference strains was not observed. However, five strains illustrate noticeable characteristics (Fig. 2). Strain DSM 16831 has a considerably low ability of adherence and no ability of invasion. In comparison to isolates characterized as common, isolate AC6827 has a low adherence and invasion, whereas isolate 134257 exposed only a low adherence. Strain DSM 13808 and isolate 05950 revealed standard adhesive characteristics but the invasion capacity was considerably higher compared to the other isolates. Correlation analysis of adherence to or invasion of endothelial cells and the number of present virulence genes revealed no correlation: (a) three virulence genes versus two virulence genes: *P*_adhesion _= 0.35, *P*_invasion _= 0.12, (b) three virulence genes versus one virulence gene: *P*_adhesion _= 0.08, *P*_invasion _= 0.19 and (c) two virulence genes versus one virulence gene: *P*_adhesion _= 0.27, *P*_invasion _= 0.81.

### Influence of cell type and cell condition on the adherence and invasion characteristics

**Figure 3:**
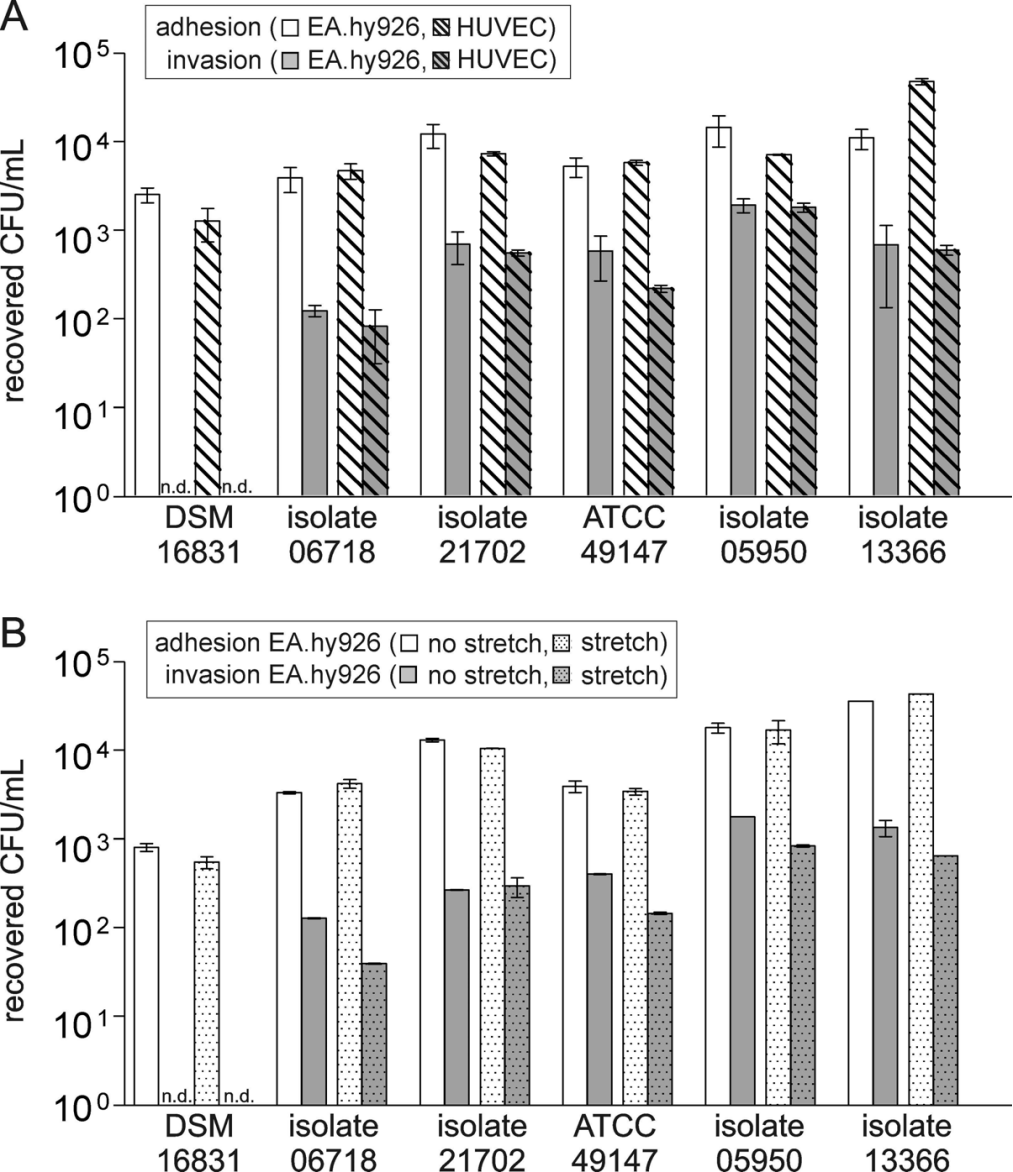
**Influence of cell type (EA.hy926/HUVEC) and cell condition (stressed/non-stressed) on the adherence and invasion characteristics of *S. gallolyticus***. (A) Adhesion to and invasion of endothelial cell lines EA.hy926 and HUVECs after infection with 1 - 9 × 10^5 ^CFU/mL of different *S. gallolyticus *strains. (B) *S. gallolyticus *strain's adhesion to and invasion of EA.hy926 with and without mechanical stretch 24 h prior to infection with 1 - 9 × 10^5 ^CFU/mL bacteria. Results were determined after a 2 h exposure followed by additional 2 h incubation in the presence of antibiotics. n.d.: not detectable.

Fig. 3 shows the adherence to and invasion of EA.hy926 and HUVECs for six bacterial strains with different adhesion and invasion potentials. The comparison of the two different cell types revealed no discrepancy between adhesion and invasion (*P *> 0.01). Therefore, the cell line EA.hy926 was chosen for further studies of *S. gallolyticus *infection of endothelial cells. As shown in Fig. 3, the adherence and invasion characteristics of *S. gallolyticus *to EA.hy926 are likewise comparable between mechanical stretched and untreated cells. However, isolates 13366, 05950, 49147 and 06718 show the tendency of a marginally decreased invasion to mechanical stretched cells.

### Binding to ECM proteins and biofilm formation

**Figure 4:**
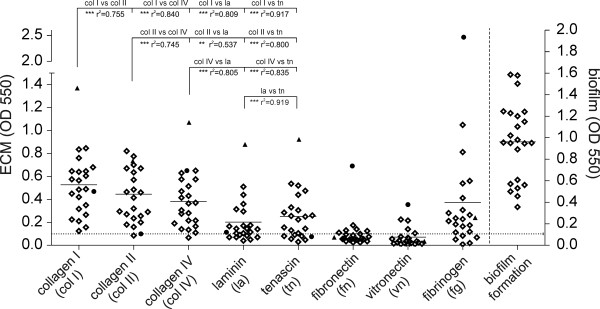
**Biofilm formation and adherence of *S. gallolyticus *strains to immobilized ECM proteins**. *Scatter plots *show the distribution of the eight ECM proteins and biofilm formation for the different strains/isolates. Individual dots represent the mean values for each strain (quadruplicate determination), the *solid horizontal *line represents the mean values of the different strains for each ECM protein tested. The *dotted horizontal *line represents the cut-off value for adherence. The *dashed vertical *line indicates the separation of the biofilm formation assay. vs: versus, ***P < 0.0001, **P < 0.001, black dot: isolate AC 7070, black triangle: isolate AC 1181.

For evaluation of the ability of *S. gallolyticus *strains to adhere to host ECM proteins, we analyzed adherence to collagen types I, II, IV, fibronectin, laminin, tenascin, vitronectin and fibrinogen (Fig. 4). Adherent bacteria were stained with CV, and parallel plating onto BHI agar confirmed the initial bacterial titer to 10^8 ^CFU/mL for all 23 strains tested. After correction with BSA negative control values, values of OD_550 _> 0.1 were considered adherent. Mean values of the three different collagen types did not differ significantly. Adherence to collagen I showed the highest values (mean 0.53 (± 0.28)), followed by collagen II (mean 0.45 (± 0.27)), collagen IV (mean 0.38 (± 0.24)), fibrinogen (mean 0.37 (± 0.52)), tenascin (mean 0.25 (± 0.21)) and laminin (mean 0.20 (± 0.19)). Accordingly, the proportion of non-adherent strains increased almost in this order. One strain was unable to adhere to collagen II and IV, whereas five strains did not adhere to fibrinogen, and seven strains did not adhere to laminin or tenascin. Binding to fibronectin and vitronectin revealed the highest proportion of non-adherent strains (fibronectin: n = 16, vitronectin: n = 18) and the observed adherence was relatively low. Individual strain correlation analysis between adherence to endothelial cells and ECM proteins showed no correlation. In contrast, analysis of the adherence of different ECM proteins showed a strong correlation (*P *< 0.0001) for the following nine protein combinations: (a) collagen I versus collagen II, IV, laminin and tenascin, respectively; (b) collagen II versus collagen IV, laminin and tenascin, respectively; (c) collagen IV versus tenascin and (d) laminin versus tenascin (Fig. 4). A correlation of moderate strength was found for the protein combination collagen IV and laminin (P < 0.001). No correlation was observed for protein combinations including fibronectin, vitronectin or fibrinogen. The ability of adherence to ECM proteins showed a tendency to cluster in certain isolates, e.g. strains with high efficiency of binding to the three different collagen types also showed a strong adherence to laminin and tenascin. Two strains exhibited a considerably higher adherence; isolate AC1181 had a high adherence to collagen I/II/IV, laminin and tenascin, whereas isolate AC7070 had a high adherence to fibrinogen, vitronectin and fibronectin.

The analysis of biofilm formation demonstrated that all strains have the capability to adhere to polystyrene surfaces and form biofilms (Fig. 4). Isolates 10672, AC1135 and strain DSM 16831 revealed the highest biofilm formation; remarkably, strain DSM 16831 had no capacity to invade cells. Correlation analysis of adherence to or invasion of endothelial cells and biofilm formation revealed no correlation. Additionally, no correlation was found between adherence to different ECM proteins and biofilm formation.

## Discussion and Conclusions

*S. gallolyticus *is an important pathogen with an underestimated relevance causing IE. The frequent changes in the taxonomy resulted in an inadequate or incorrect identification of the causative pathogens, because non-experts were often not aware of the new nomenclature (e.g. [42]). In contrast to other streptococci, little is known about virulence factors and pathogenesis. The adherence of circulating bacteria to damaged heart tissues and subsequent colonization and persistence of bacteria are the crucial factors in streptococcal IE. The prevention of tissue colonization, with special attention to targeting therapy against ECM-binding, potentially provides a promising alternative in human medicine [43]. Therefore, we analyzed the factors which contribute to *S. gallolyticus *adhesion and invasion in IE using an experimental *in vitro *cell culture model. Investigation of the adhesion to ECM proteins identified or confirmed putative adhesive sites on the endothelial cell surface. Additionally, virulence factors were detected and biofilm formation was analyzed in order to identify different strain characteristics.

Most *S. gallolyticus *strains tested in this study adhere to and invade endothelial cells. The diversity in adhesion and invasion characteristics appears considerably higher for invasion. Strain DSM 16831 exclusively demonstrated no invasive capability. Invasion was also not induced using higher concentrations of bacteria, usage of primary endothelial cells or mechanical stretched cells. In contrast, strain DSM 13808 present a considerably high invasion. The distinct behaviour of these two strains may be due to the fact that they were the only strains tested that were isolated from non-human sources. In general, the observed differences may reflect distinctions in the bacterial equipment with virulence factors or gene expression of virulence factors. We have shown that isolate represent a different distribution of the virulence-associated genes *gtf*, *fimB *and *pilB*. However, the presence of a putative virulence gene does not necessarily indicate expression. For example, Stipp *et al. *observed variability at different phases of planktonic growth in *gtfB *and *gtfC *gene expression [44]. Furthermore, the gene integrity has to be proven. To correlate virulence with the expression of *gtf*, *fimB *and *pilB*, these factors have to be deleted by the construction of knock-out mutants to determine difference in the ability to form biofilms, the adherence to and invasion of host cells and the adherence to ECM proteins. This will show the impact of these factors on binding to host cells and most likely correlate with the bacterial potential to cause IE. In addition, the determined virulence factors reflected only a small proportion of the presumably high quantity of possible virulence factors in *S. gallolyticus*. Accordingly, the absence of a correlation between the potential to adhere to as well as to invade cells and the number of the existing putative virulence genes most likely could also be explained by these reasons.

The role of biofilm formation in IE remains ambiguous. Several studies demonstrated an association between biofilm formation and streptococcal IE [45-47], whereas another study indicated that the ability to form biofilms *in vitro *is not associated with endocarditis virulence [30]. The results of our study support the lack of association between biofilm formation and adherence to or invasion of endothelial cells and adherence to ECM proteins.

Most IE patients have valve abnormalities, resulting in the exposure of ECM proteins, the production of tissue factor and the deposition of fibrin and platelets promoting bacterial colonization. Streptococcal adherence to endothelial matrix proteins has previously been shown to be an important factor for the infection of host tissues [32-37,48]. Recently, Sillanpää *et al. *analyzed endocarditis-derived human isolates of *S. gallolyticus *and, according to the results obtained in our study, binding to collagen I was found to be the most common phenotype, followed by collagen type IV, fibrinogen and fibronectin [2]. In contrast, both studies revealed a weak binding to fibronectin, which is contradictory to studies observing a direct connection between adherence to fibronectin and the applicability of *S. sanguis *to induce IE [49]. This observation possibly indicates a different pathogenesis of *S. gallolyticus *IE. Interestingly, a study of animal isolates of *S. gallolyticus *revealed no adherence to collagen I [12]. Further analysis of the draft genome sequence of an ECM protein-adherent *S. gallolyticus *strain by Sillanpää *et al. *revealed 11 predicted LPXTG-type cell-wall-anchored proteins with characteristics of MSCRAMMs (microbial surface components recognizing adhesive matrix molecules), including the "adhesin to collagen of the *S. bovis *group" (*acb*) gene [50]. Remarkably, a recombinant Acb protein showed high affinity binding to immobilized collagen. Cell surface expression of Acb correlated with the presence of *acb *and collagen adherence of different isolates. Therefore, the authors concluded that the high prevalence of this collagen-binding MSCRAMM among *S. gallolyticus *may play an important role in the predominance of this subspecies in *S. bovis *complex endocarditis.

The endothelial cell line EA.hy926 displays highly differentiated characteristics of human vascular endothelial [51] whereas primary endothelial cells such as HUVECs presumably provide the most accurate cell type based reflection of the *in vivo *situation. However, we observed no difference in the adhesion and invasion characteristics of *S. gallolyticus *using these two cell lines. Consequently, the usage of endothelial cell lines seems to be an equivalent experimental *in vitro *model, with the major advantage of easier handling compared to primary cells. Nonetheless, it has to be noted that cell monolayers of either cell lines or primary cells only provide a two-dimensional model, whereas the *in vivo *situation in tissue is three-dimensional.

The intact endothelium is usually resistant to colonization by streptococci [18]. In the present study, mechanical stress of endothelial monolayer does not increase the proportion of adherent or invasive bacteria. This data is an indication for active colonization of valve tissue by *S. gallolyticus*. However, the results have to be interpreted with caution. We cannot exclude the possibility that mechanical stretch does not significantly increase the degree of stress on the potentially damaged cell monolayer. In addition, monolayers probably do not exhibit a physically intact endothelium since two-dimensional cultivation or contact-inhibition perhaps affected the endothelial cells. Therefore, further studies are warranted to figure out the degree of monolayer integrity and the dimension of cell damage before and after mechanical stretch.

The data of our study demonstrates that there is no evidence for the correlation between adherence to or invasion of endothelial cells, the adherence of bacteria to ECM proteins and biofilm formation. Therefore several other factors have to be investigated to determine their role in the infection of endothelial cells by *S. gallolyticus *isolates. These factors might include the capsule structure [52], interaction with cell surface glycosaminoglycans [53], presence of fimbriae or production of toxins [15]. It has been shown that *S. gallolyticus *is capable to produce capsular material [15] and the amount of capsule produced most likely influence the capacity to adhere to the cells. Hence, analysis of further pathomechanisms beneath adhesion, invasion and biofilm formation characteristics as well as the identification of further putative virulence genes is crucial for a better understanding of the mechanisms of *S. gallolyticus *infection. Our future investigations will address the transcriptional analysis of known virulence factors, the identification and characterization of further putative virulence genes by sequencing the whole genome of *S. gallolyticus *and knock-out mutation experiments to determine the influence of individual gene function in the pathogenesis of IE. As a result of the distinct behaviour of the isolates from non-human sources, we will also focus on the comparison of human and animal isolates to further elaborate potential differences in infection mechanisms. The specific clinical association with gastrointestinal neoplasia due to *S. bovis *biotype I or *S. gallolyticus*, respectively [7-9] strongly imply that *S. gallolyticus *enter the human body via the gastrointestinal tract through sites with decreased intestinal barrier function such as colonic malignancies. Unfortunately, a correlation between the number of existing virulence genes, biofilm formation, invasion and adhesion characteristics with the presence or absence of colonic malignancies can barely be created with the small number of available patient data at present. Indeed, the bacterial translocation is the first important step in the development of IE before colonizing the endothelium, and mechanisms of adherence to and invasion of epithelial cells play an important role during this initial phase of infection. Therefore, our future investigations will also address this important mechanism to potentially disclose clues on specific features of individual *S. gallolyticus *strains.

In conclusion, this is the first description of *S. gallolyticus *adhesion to and invasion of human endothelial cells. The established *in vitro *model provides a convenient system to evaluate differences in the virulence characteristics of different strains. Binding to ECM proteins and biofilm formation provide additional information for strain characterization. The first identification of a possible pilus-associated gene in *S. gallolyticus *supplemented the so far limited availability of possible virulence factors. This study provides important initial characterization of variability and behaviour of the as yet barely analyzed endocarditis pathogen *S. gallolyticus*.

## Abbreviations

BHI: brain heart infusion; CV: crystal violet; DMEM: Dulbecco's modified essential medium; DPBS: Dulbecco's phosphate buffered saline; ECM: extracellular matrix; HUVECs: human umbilical vein cells; IE: infectious endocarditis; MSCRAMMs: microbial surface components recognizing adhesive matrix molecules; TS: tryptone soya.

## Authors' contributions

TV carried out the adhesion and invasion studies and drafted the manuscript. DH carried out the molecular genetic studies, the biofilm formation assays and helped to draft the manuscript.

KK conceived and designed the study and revised the manuscript critically for important intellectual content. JD supervised the study and participated in its design and coordination, analyzed and interpreted data and revised the manuscript critically for important intellectual content. All authors read and approved the final manuscript.
